# MRI-Based Radiomics and Artificial Intelligence for Prediction of Recurrence and Prognostic Outcomes in Oral Tongue Squamous Cell Carcinoma: A Systematic Review with Functional Meta-Synthesis

**DOI:** 10.3390/medsci14020332

**Published:** 2026-06-19

**Authors:** Carlos M. Ardila, Eliana Pineda-Vélez, Anny M. Vivares-Builes, Alejandro I. Díaz-Laclaustra

**Affiliations:** 1 Department of Periodontics, Saveetha Dental College, and Hospitals, Saveetha Institute of Medical and Technical Sciences, Saveetha University, Chennai 600077, India; 2 Basic Sciences Department, Biomedical Stomatology Research Group, Faculty of Dentistry, Universidad de Antioquia U de A, Medellín 050010, Colombia; eliana.pineda@uam.edu.co (E.P.-V.); anny.vivares@uam.edu.co (A.M.V.-B.); 3 Faculty of Dentistry, Institución Universitaria Visión de las Américas, Medellín 050040, Colombia; 4 Department of Basic Sciences, Faculty of Dentistry, Universidad de Antioquia, Medellín 050010, Colombia; isbet.diaz@udea.edu.co

**Keywords:** oral tongue squamous cell carcinoma, magnetic resonance imaging, radiomics, artificial intelligence, prognosis, systematic review, functional meta-synthesis

## Abstract

Background/Objectives: Oral tongue squamous cell carcinoma (OTSCC) remains clinically challenging because conventional clinicopathological markers do not fully explain variability in recurrence and survival. This systematic review and functional meta-synthesis aimed to identify and critically appraise studies using preoperative magnetic resonance imaging (MRI)-based radiomics, artificial intelligence (AI), deep learning, or quantitative MRI-derived models to predict recurrence and prognostic outcomes in OTSCC. Methods: PubMed, Scopus, and Embase were searched from inception to March 2026. Eligible studies included prognostic model investigations in adults with OTSCC or primary tongue cancer without reported base-of-tongue/oropharyngeal involvement, undergoing preoperative MRI and surgery, with recurrence- or survival-related follow-up. The primary synthesis was a functional meta-synthesis; pooling was not performed because studies were not sufficiently comparable. Results: Seven retrospective studies were included, with a summed descriptive sample of 1287 participants. The evidence base was heterogeneous in MRI sequences, segmentation workflows, model architecture, validation strategy, and endpoint definition. Functional meta-synthesis identified four domains: direct recurrence-oriented modeling, broader prognostic stratification, reported incremental or complementary value over clinical frameworks, and translational maturity/technical implementation. Several studies reported associations between MRI-derived signatures and recurrence- or survival-related outcomes, but findings were interpreted narratively because of differences in primary endpoints, imaging features, model design, validation methods, and outcome definitions. Most studies were judged at high overall risk of bias, and certainty of evidence ranged from low to very low. Conclusions: MRI-based radiomics and AI show preliminary promise for prognostic stratification in OTSCC, particularly recurrence-related risk refinement, but current evidence remains limited by retrospective design, heterogeneity, sparse external validation, and low certainty.

## 1. Introduction

Oral tongue squamous cell carcinoma (OTSCC) is among the most clinically relevant malignancies of the oral cavity because of its invasive growth pattern, frequent cervical spread, and non-negligible risk of recurrence even after apparently adequate primary treatment [1,2]. Recent epidemiologic analyses indicate that tongue cancer continues to represent an important public health burden, while contemporary clinical series confirm that survival remains strongly conditioned by tumour site, stage, and subsequent disease control [2,3]. Within oral squamous cell carcinoma, the tongue subsite deserves particular attention because its complex muscular architecture facilitates deep infiltration and loco-regional extension, making prognostic assessment especially challenging [4,5].

The current prognostic framework in OTSCC is still anchored mainly in clinicopathological variables such as tumour size, depth of invasion (DOI), nodal status, extranodal extension, perineural invasion, margin status, and stage grouping [4,5,6]. DOI, in particular, has become central to contemporary prognostic stratification because it helps estimate the risk of occult nodal disease, recurrence, and survival outcomes, and the incorporation of DOI and extranodal extension into the 8th American Joint Committee on Cancer (AJCC) staging system has improved prognostic discrimination compared with earlier editions [4,6]. Nevertheless, even these established markers do not fully capture intratumoural heterogeneity or adequately explain the variability in recurrence and survival observed among patients with seemingly similar clinicopathological profiles [2,4,5,6]. This limitation is especially relevant in OTSCC, where local control, regional recurrence, and disease-specific death can diverge substantially despite similar conventional staging [2,3,5].

Magnetic resonance imaging (MRI) is the reference imaging modality for local assessment of OTSCC because of its superior soft-tissue contrast, multiplanar capability, and utility for estimating submucosal spread and DOI before surgery [4]. However, conventional MRI interpretation remains predominantly qualitative and observer-dependent. Although preoperative MRI can improve anatomic characterization and treatment planning, it does not by itself convert imaging into a robust individualized prognostic tool [4]. In parallel, biopsy-based assessment, while indispensable for diagnosis, may incompletely represent the full biological heterogeneity of the tumour. These limitations have driven interest in quantitative imaging strategies capable of extracting latent phenotypic information from routine radiological studies [7,8].

Radiomics and artificial intelligence (AI) have emerged as promising approaches to address this unmet need. Radiomics enables the high-throughput extraction of quantitative features related to intensity, texture, shape, and spatial heterogeneity from standard-of-care images, whereas AI and machine-learning methods may integrate these features with clinical data to generate predictive and prognostic models [7,8]. Across oncology, these methods have been increasingly investigated as non-invasive biomarkers for outcome prediction, and recent head and neck literature suggests that MRI-based radiomics may provide clinically meaningful prognostic information beyond conventional variables, although concerns remain regarding reproducibility, reporting quality, feature stability, and external validation [8,9,10]. A recent meta-analysis in head and neck cancers further suggested that radiomics may have prognostic value overall, particularly when integrated with clinical predictors, but also underscored substantial methodological heterogeneity across studies [10].

Against this background, a small but growing body of primary research has begun to explore MRI-based radiomics, deep learning, and quantitative MRI-derived modelling in OTSCC, or in surgically treated primary tongue cancer cohorts without reported base-of-tongue or other oropharyngeal involvement. These studies have assessed recurrence-oriented endpoints and survival-related outcomes using preoperative MRI sequences, including contrast-enhanced T1-weighted imaging, T2-weighted imaging, diffusion-weighted imaging, and apparent diffusion coefficient maps. However, the available evidence remains fragmented across retrospective single-centre model-development studies, with marked variation in patient populations, MRI acquisition protocols, segmentation workflows, feature engineering pipelines, model families, outcome definitions, and validation strategies [9,10,11]. Such heterogeneity has made it difficult to determine how close the field is to genuine clinical translation, which modelling strategies appear most credible, and whether any quantitative synthesis is methodologically defensible.

An additional reason for a focused synthesis is that the relevant review literature, although expanding, does not answer the present question directly. Existing reviews have addressed AI for diagnosis of tongue cancer, preoperative prediction of occult nodal metastasis and depth of invasion in OTSCC, and broader oral-cancer AI applications; however, no review has specifically synthesized MRI-based radiomics and AI models for recurrence and prognostic outcomes in OTSCC [12,13,14]. Jeong et al. [12] reviewed AI for the diagnosis of tongue cancer, but their emphasis was diagnostic imaging rather than recurrence or survival modelling. Ho et al. [13] focused on AI-based preoperative prediction of occult lymph node metastasis and DOI in OTSCC, which is highly relevant but conceptually distinct from recurrence- and survival-oriented prognostic modelling. Mohideen et al. [14] synthesized AI and radiomics for oral-cancer lymph node metastasis prediction and tumour grading across broader oral-cancer cohorts, again addressing a different target domain. Earlier reviews by Lenze et al. [15], Almangush et al. [16], and Voizard et al. [11] examined recurrence risk by age, prognostic biomarkers, and preoperative DOI measurement in OTSCC, respectively, but none specifically addressed MRI-based radiomics and AI models designed for recurrence and other prognostic endpoints [11,15,16].

The knowledge gap, therefore, is not merely the absence of primary studies but the absence of a review specifically organized around the prognostic role of preoperative MRI-derived computational modelling in OTSCC. A focused systematic review is justified because it can clarify which outcomes have been modelled, which MRI sequences and segmentation strategies have been used, whether hybrid clinical–radiomic models report complementary value over conventional predictors, and whether the existing evidence is sufficiently comparable to support quantitative synthesis. This evidence-synthesis question should be distinguished from primary AI model development, which requires large, standardized datasets for training, tuning, external validation, and prospective testing. Just as importantly, such a review can identify the methodological limitations that currently constrain reproducibility and clinical implementation, including limited external validation, inconsistent endpoint definitions, anatomical-site ambiguity in some studies, and variability in imaging-analysis workflows [7,8,9,10].

Accordingly, the present systematic review with functional meta-synthesis was conducted to systematically identify, describe, and critically appraise original studies that used MRI-based radiomics, AI, deep learning, or quantitative MRI-derived models to predict recurrence and prognostic outcomes in OTSCC. The review also aimed to summarize model characteristics, MRI sequences, segmentation approaches, validation strategies, reported predictive performance, and the anatomical site definitions used in the included studies. By doing so, it seeks to fill a clinically relevant and methodologically well-defined gap in the literature and to clarify the current evidence base for MRI-based computational biomarkers within precision oncologic imaging in OTSCC.

## 2. Materials and Methods

This systematic review with functional meta-synthesis was designed and reported in accordance with the Preferred Reporting Items for Systematic Reviews and Meta-Analyses (PRISMA) 2020 statement [17]. The protocol was registered in the International Prospective Register of Systematic Reviews (PROSPERO) (CRD420261375910). The PROSPERO record is publicly accessible, and no major deviations from the prespecified protocol were made in the revised manuscript. Quantitative pooling was considered only as a conditional methodological option when clinically and methodologically comparable estimates were available; however, no quantitative pooling was performed. The review focused on original studies evaluating preoperative MRI-based radiomics, artificial intelligence, deep learning, or quantitative MRI-derived prognostic models in OTSCC, with emphasis on recurrence-related and survival-related outcomes.

### 2.1. Eligibility Criteria

Eligibility was defined according to a PICOS (Participants, Index model/exposure, Comparators, Outcomes, and Study design) framework aligned with the protocol. Participants were adults with pathologically confirmed OTSCC, defined as squamous cell carcinoma of the oral/mobile tongue, who underwent preoperative MRI followed by surgical treatment and for whom recurrence-related or survival-related follow-up data were available. Eligible index models or exposures included MRI-based radiomics models, machine learning models, deep learning models, clinicoradiomic nomograms, and quantitative MRI-derived prognostic models built from preoperative MRI sequences such as contrast-enhanced T1-weighted imaging, T2-weighted imaging, diffusion-weighted imaging, apparent diffusion coefficient maps, multiparametric MRI, or MRI-derived peritumoural modelling. These approaches were not considered technically equivalent; rather, they were grouped because they shared a common prognostic premise: the use of preoperative MRI-derived quantitative or computational information to model recurrence-related or survival-related outcomes in OTSCC.

Comparators were not mandatory for study inclusion. When present, acceptable comparators included clinical models alone, clinicopathological predictors, alternative MRI-derived models, alternative MRI sequences, non-radiomic models, and manual versus automated workflows. Original prognostic or predictive model studies were eligible, including retrospective or prospective cohort studies, model-development studies, model-validation studies, and combined development-validation studies published as full-text articles.

Studies were excluded if they enrolled pediatric populations, animals, in vitro models, mixed head and neck cohorts without extractable oral/mobile tongue-specific data, base-of-tongue or other oropharyngeal tumors without extractable oral/mobile tongue-specific data, or non-tongue oral cavity subsites only. We also excluded studies based exclusively on computed tomography (CT), positron emission tomography (PET), cone-beam computed tomography (CBCT), ultrasound, histopathology, genomics, blood biomarkers, or clinical variables alone; purely diagnostic or staging studies without recurrence or prognostic outcomes; studies focused only on cervical lymph node metastasis unless recurrence risk or survival-related outcomes were also explicitly modelled; and non-original reports such as reviews, editorials, letters, conference abstracts without full reports, case reports, case series without a prognostic model, and technical imaging papers without clinical outcomes.

### 2.2. Information Sources and Search Strategy

A systematic literature search was undertaken in PubMed (MEDLINE), Scopus, and Embase from database inception to March 2026, without language or date restrictions. Only published studies were sought. The electronic search was supplemented by backward citation searching of all included papers, forward citation tracking of eligible studies, and targeted checks of relevant registers or author correspondence when clarification of study eligibility or data reporting was necessary.

Search strategies were developed by combining controlled vocabulary when applicable and free-text terms related to tongue cancer, MRI, radiomics, artificial intelligence, deep learning, recurrence, and prognosis. During piloting, database-specific syntax was iteratively refined to maximize stable retrieval and platform compatibility. The complete final search strategies for PubMed, Scopus, and Embase are presented in Supplementary Table S1.

### 2.3. Selection Process

All records were imported into a reference management environment and deduplicated before screening. Two reviewers independently screened titles and abstracts against the predefined eligibility criteria. Full texts of potentially relevant records were then reviewed independently by the same reviewers. Any disagreement was resolved first by discussion and, when consensus was not reached, by consultation with a third reviewer. Reasons for exclusion at the full-text stage were documented to ensure reproducibility and transparency, consistent with PRISMA recommendations [17].

### 2.4. Data Collection Process

Data extraction was performed independently by two reviewers using a piloted standardized extraction form derived from the protocol. When required information was incompletely reported, study authors were to be contacted for clarification. Discrepancies between extractors were resolved by consensus, with arbitration by a third reviewer when necessary.

The extraction form was structured to capture bibliographic details, study setting, country, sample size, patient and tumour characteristics, MRI platform and sequences, segmentation approach, feature extraction and feature selection pipeline, model family, comparator structure when present, outcome definitions, validation strategy, performance metrics, effect estimates, and principal conclusions.

### 2.5. Data Items

The following variables were prespecified for collection from each study: authorship, year of publication, country, study design, sample characteristics, tumour site definition, MRI protocol and sequence type, field strength, segmentation method, region-of-interest strategy, peritumoural modelling when applicable, feature extraction software or pipeline, feature-selection strategy, model type, predictors retained in the final model, comparator model(s), outcome definition(s), internal or external validation characteristics, discrimination and calibration results, survival-related effect estimates, and information relevant to synthesis domains such as degree of automation and incremental value over clinical models.

### 2.6. Study Risk of Bias Assessment

Risk of bias and applicability were assessed independently by two reviewers using the Prediction model Risk Of Bias ASsessment Tool (PROBAST), which was specifically developed for diagnostic and prognostic prediction model studies [18]. PROBAST evaluates four domains: participants, predictors, outcome, and analysis. Domain-level and overall judgments were assigned following the signaling questions and interpretive guidance of the tool. Discrepancies were resolved by discussion, and consensus ratings were tabulated for presentation.

Because several included studies were expected to involve machine learning or deep learning components, key reporting features relevant to model transparency and reproducibility were also summarized narratively in light of the Transparent Reporting of a multivariable prediction model for Individual Prognosis Or Diagnosis plus Artificial Intelligence (TRIPOD + AI) guidance, without treating reporting quality as a substitute for risk of bias assessment [19].

### 2.7. Certainty of Evidence

The certainty of the evidence was planned to be judged cautiously at the outcome level using a Grading of Recommendations Assessment, Development and Evaluation (GRADE)-informed approach adapted for prognosis research [20,21]. The assessment considered risk of bias, inconsistency, indirectness, imprecision, and potential publication bias, together with issues especially relevant to prediction research, such as external validation, reproducibility, and stability of estimates across clinically comparable studies. Overall certainty ratings were intended to be expressed as high, moderate, low, or very low, recognizing that small, heterogeneous prognostic model studies frequently warrant cautious interpretation.

### 2.8. Data Synthesis and Statistical Analysis

All included studies were first synthesized descriptively. Study characteristics, MRI protocols, model families, comparator structures, validation designs, and reported performance metrics were summarized in evidence tables and narratively contrasted across studies. Given the anticipated heterogeneity in endpoints, imaging workflows, validation strategies, and model architecture, the principal analytic strategy was a functional meta-synthesis.

The functional meta-synthesis was conducted through a transparent and reproducible multistep procedure adapted from thematic synthesis principles [22]. First, all included studies were read in full and entered into an analytic matrix containing their main objective, target outcome, MRI sequence(s), segmentation strategy, modelling approach, validation design, and headline results. Second, two reviewers independently assigned preliminary descriptive codes to each study, focusing on the dominant clinical purpose of the model, the type of prognostic endpoint addressed, and the methodological role played by the MRI-derived signature. Third, these initial codes were compared and iteratively consolidated into a coding framework through constant comparison across studies. Fourth, each study was assigned to one dominant functional domain according to prespecified decision rules: the authors’ primary stated aim, the principal modelled endpoint, and the main interpretive emphasis in the results and discussion sections were prioritized over secondary analyses. Studies in which lymph node metastasis (LNM) was the primary modelling target but disease-free survival (DFS), overall survival (OS), or recurrence-related associations were reported secondarily were not classified as direct recurrence- or survival-prediction studies; instead, they were retained as indirect prognostic evidence and interpreted separately within the functional synthesis. Studies could also receive secondary tags when they contributed meaningfully to more than one domain.

The functional domains were defined to include, where applicable: recurrence prediction; survival or prognostic stratification; integration of MRI-derived signatures with clinicopathological variables; contribution of peritumoural or depth-of-invasion-related information; and degree of automation within the imaging-analysis workflow. After domain allocation, evidence was synthesized within and across domains by comparing MRI sequences, segmentation approaches, feature pipelines, model architecture, validation strategy, and reported predictive performance. A cross-domain interpretive matrix was then used to identify recurring methodological patterns, convergent findings, and unresolved areas of heterogeneity.

Quantitative pooling was considered only if studies reported clinically and methodologically comparable estimates for the same primary prognostic endpoint, model target, and validation context. Priority would be given to hazard ratios for recurrence-related or survival-related outcomes only when pooling was clinically and methodologically justified. If quantitative synthesis was feasible, random-effects models were planned because heterogeneity was expected, and inference with few studies would be interpreted conservatively with reference to the Hartung–Knapp–Sidik–Jonkman approach [23]. Statistical heterogeneity would be examined using I^2^ and related measures [24]. Performance metrics such as AUC or C-index were not to be pooled automatically; they would only be summarized quantitatively if outcome definitions, modelling targets, and validation contexts were sufficiently comparable. If comparability was inadequate, findings would remain narratively synthesized. Assessment of publication bias or small-study effects was planned only if at least 10 sufficiently comparable studies were available for a given quantitative synthesis; otherwise, reporting bias would be addressed narratively.

## 3. Results

### 3.1. Study Selection Process

**Figure 1:**
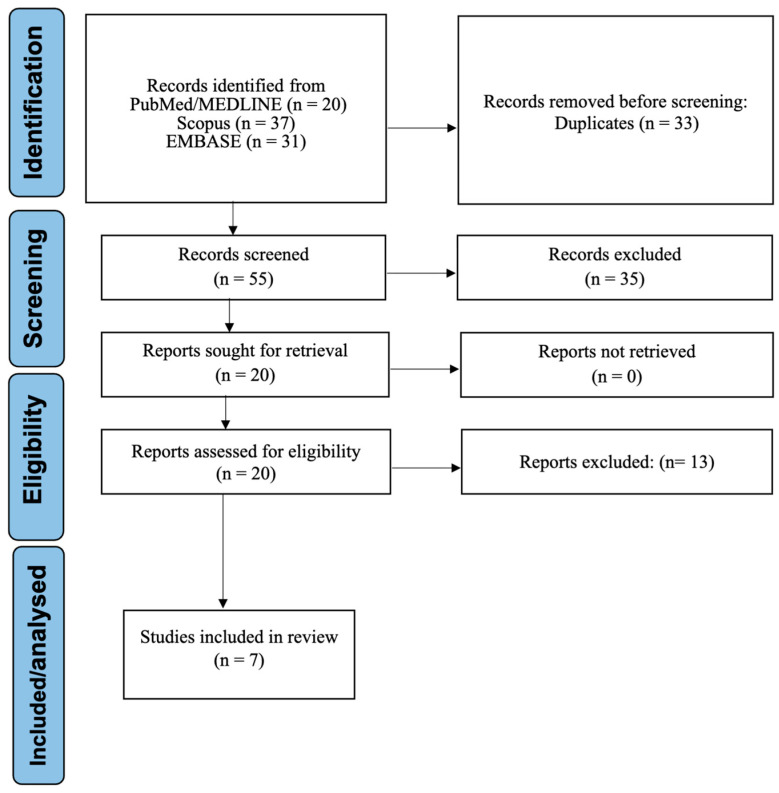
PRISMA flow diagram showing the study selection process.

The study selection process is summarized in the PRISMA flow diagram (Figure 1). A total of 88 records were identified through database searching. After removal of 33 duplicates, 55 unique records remained for title and abstract screening. Of these, 35 were excluded because they did not address MRI-based prognostic modeling in oral tongue squamous cell carcinoma, did not report recurrence- or survival-related outcomes, focused on diagnosis or nodal prediction without prognostic linkage, included mixed head and neck cohorts without extractable oral/mobile tongue-specific data, or corresponded to non-eligible publication types. Twenty articles underwent full-text assessment. After detailed evaluation, 13 studies were excluded, most commonly because they focused primarily on lymph node metastasis or nodal staging without recurrence- or survival-related prognostic endpoints, were not based on MRI-derived radiomics/AI-oriented prognostic modeling, were not original primary studies, did not provide extractable oral/mobile tongue-specific prognostic data, or included base-of-tongue tumors without separate OTSCC-specific results. Seven studies fulfilled all eligibility criteria and were included in the systematic review and functional meta-synthesis. Supplementary Table S2 presents the 13 excluded full-text articles with reasons for exclusion.

### 3.2. Descriptive Characteristics of Included Studies

A total of seven retrospective studies were included in this review, published between 2022 and 2026 and conducted in two countries, China and Italy [25,26,27,28,29,30,31]. Across the included studies, the total reported sample comprised 1287 participants; this number was interpreted descriptively and not as a pooled dataset for AI model development or validation. The evidence base consisted of independent retrospective cohorts with different MRI protocols, segmentation strategies, feature-extraction pipelines, model architectures, endpoints, and validation procedures. It included recurrence- or prognosis-oriented model-development studies [26,28,29,30], one multicentric external validation study of previously published MRI-radiomic models [27], one two-center automated deep-learning study with internal and external testing cohorts [25], and one LNM-focused study with training/testing cohorts and secondary DFS/OS prognostic associations [31]. The main descriptive characteristics of the included studies are summarized in Table 1.

Overall, the included studies showed substantial heterogeneity in sample size, modeling strategy, MRI sequences, and validation framework [25,26,27,28,29,30,31]. Although most studies evaluated manually delineated tumour volumes or regions of interest, one study implemented a fully automated pipeline based on YOLOv8 tumour detection followed by deep-learning prediction [25]. Multiparametric MRI was common, with recurrent use of contrast-enhanced T1-weighted imaging, T2-weighted imaging, diffusion-weighted imaging, and apparent diffusion coefficient maps [25,27,28,29,30], whereas some studies used more specific approaches, such as peritumoural modeling on T2-weighted imaging [31] or post-contrast MRI-derived difference volumes [26].

The studies also differed in their outcome structure and validation strategies. Some focused more directly on recurrence-oriented endpoints, whereas others emphasized broader prognostic outcomes, including survival-related measures or composite adverse events. Internal or temporal validation was reported in most studies, while explicit external validation was clearly represented by the multicentric validation study and the two-center deep-learning framework [25,27]. Taken together, these characteristics indicate that the current evidence base is methodologically diverse and still evolving, particularly with respect to automation, sequence selection, and validation rigor.

### 3.3. Outcomes

The main recurrence-related outcomes specified in the protocol were collectively represented across the included studies, although individual studies contributed unevenly to the different endpoints. The contribution of each included study to the main and additional outcomes is detailed in Table 2.

Recurrence-oriented outcomes were most directly represented by studies focused on loco-regional recurrence, locoregional recurrence-free survival, disease-free survival, and recurrence risk [26,27,29,30]. Two additional studies, Ren et al. [25] and Wang et al. [31], primarily addressed lymph node metastasis prediction but reported secondary DFS- or OS-related prognostic associations. These studies were therefore retained as indirect prognostic evidence but were not treated as direct recurrence- or survival-modeling studies. Taken together, these studies supported the predefined outcome framework while also illustrating variability in how recurrence-related prognosis was operationalized across the literature. Accordingly, interpretation of the main recurrence- and survival-oriented findings was primarily anchored in studies in which these outcomes were the principal modelling target, whereas LNM-focused studies were used only to contextualize secondary prognostic associations.

The prespecified performance measures were also variably reported across studies. Discrimination was described using the area under the receiver operating characteristic curve (AUC) and/or concordance index (C-index) in most articles, whereas classification-oriented metrics such as accuracy, sensitivity, and specificity were more common in recurrence or nodal-risk classification models. Hazard ratios were mainly available in studies that linked MRI-derived signatures with time-to-event outcomes, and calibration procedures were reported more often in nomogram-based or validation-focused analyses. Overall, the reported metrics were broadly consistent with the planned outcome framework, but their diversity confirmed substantial methodological heterogeneity.

The additional outcomes planned in the protocol were also well represented. Overall survival was reported in several studies, cause-specific mortality was addressed in a smaller subset, and validation characteristics ranged from internal or temporal validation to explicit external multicentric testing [25,27,28,29,30,31]. Segmentation strategy and sequence-specific performance also emerged as important secondary findings. Manual region-of-interest/volume-of-interest (ROI/VOI) delineation predominated in most studies, whereas automated tumour detection was implemented in only one investigation [25]. Likewise, the prognostic contribution of MRI sequences differed across studies, with some identifying ADC or CE-T1 as the best-performing sequence, and others excluding ADC- or DWI-derived signatures from final models because of limited reproducibility or discriminatory value. These findings indicate that the clinical utility of MRI-derived prognostic signatures remains closely linked to sequence choice, segmentation workflow, and validation strategy.

Finally, several studies evaluated whether MRI-derived models reported complementary prognostic performance beyond clinical or clinicopathological variables alone [25,26,27,28,29,31]. In most cases, combined models showed better or complementary performance compared with selected clinical or imaging-only comparators. However, because comparator structures were heterogeneous and not always aligned with fully optimized contemporary clinicopathological frameworks, these findings should be interpreted as preliminary evidence of possible complementary value rather than as proof of established clinical utility.

### 3.4. Functional Meta-Synthesis

Although each study was assigned a dominant functional domain during the analytic process, several also contributed secondarily to adjacent domains; therefore, the synthesis below emphasizes both primary allocation and cross-domain interpretive overlap.

**Figure 2:**
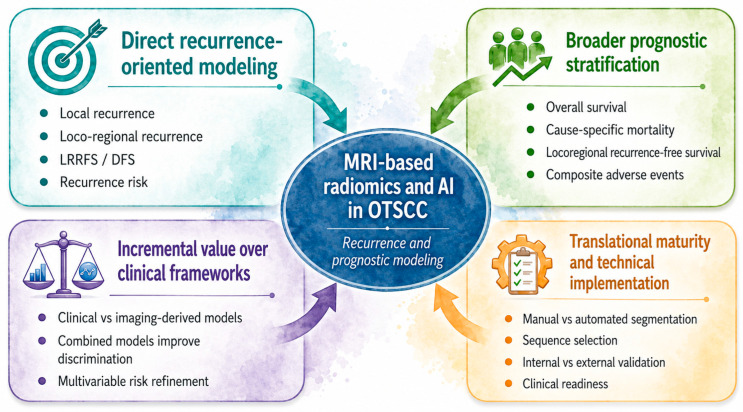
Functional meta-synthesis of MRI-based radiomics and artificial intelligence for recurrence and prognostic modeling in oral tongue squamous cell carcinoma. The figure summarizes the four functional domains identified across the included studies. Direct recurrence-oriented modeling was represented mainly by Vidiri et al. [26], Mossinelli et al. [29], Tagliabue et al. [27], and Cai et al. [30]. Broader prognostic stratification was reflected by Yao et al. [28], Mossinelli et al. [29], and Tagliabue et al. [27], with Ren et al. [25] and Wang et al. [31] contributing indirect prognostic information through secondary DFS/OS associations from LNM-focused models. Reported incremental or complementary value over clinical frameworks was summarized in Ren et al. [25], Vidiri et al. [26], Tagliabue et al. [27], Yao et al. [28], Mossinelli et al. [29], and Wang et al. [31], with interpretation limited by heterogeneous clinical comparators and validation strategies. Translational maturity and technical implementation encompassed segmentation workflow, sequence selection, and validation strategy across all included studies [25,26,27,28,29,30,31], with the highest level of automation reported by Ren et al. [25] and explicit external validation reported by Tagliabue et al. [27] and Ren et al. [25].

Inductive coding of the included studies identified four interrelated functional domains: (1) direct recurrence-oriented modeling, (2) broader prognostic stratification, (3) incremental value of MRI-derived signatures over clinical frameworks, and (4) translational maturity and technical implementation. These domains are summarized in Figure 2.

The first domain, direct recurrence-oriented modeling, comprised studies in which recurrence-related endpoints were the principal analytic target. Vidiri et al. [26] focused specifically on loco-regional recurrence prediction using MRI radiomics. Mossinelli et al. [29] modeled loco-regional recurrence together with survival outcomes, while Tagliabue et al. [27] externally validated MRI-radiomic models for locoregional recurrence-free survival and related prognostic endpoints. Cai et al. [30] also belonged to this domain by developing a prognostic model based on the apparent diffusion coefficient ratio to predict recurrence and disease-free survival. Collectively, these studies indicate that preoperative MRI-derived signatures may capture recurrence vulnerability before surgery, although the exact operationalization of recurrence differed across studies.

The second domain, broader prognostic stratification, encompassed studies in which recurrence was embedded within wider outcome frameworks or accompanied by survival-oriented prediction. Yao et al. [28] developed a clinical–radiomic nomogram for overall survival and adverse-event risk. Mossinelli et al. [29] and Tagliabue et al. [27] also contributed to this domain by incorporating overall survival, cause-specific mortality, and locoregional recurrence-free survival into their prognostic frameworks. Wang et al. [31] and Ren et al. [25] primarily addressed lymph node metastasis prediction, but both reported secondary associations with disease-free survival or overall survival. These findings were interpreted as indirect prognostic signals rather than as evidence from models primarily designed or validated for recurrence or survival prediction. Functionally, this domain shows that the literature has moved beyond isolated recurrence prediction toward more comprehensive preoperative risk stratification models, although the available evidence remains heterogeneous in endpoint definition and model target.

The third domain, reported incremental or complementary value of MRI-derived signatures over clinical frameworks, captured studies that examined whether radiomics, deep learning, or quantitative MRI-derived signatures improved or complemented prognostic performance beyond clinical or clinicopathological variables alone. This pattern was particularly evident in Yao et al. [28], Wang et al. [31], and Ren et al. [25], where combined models outperformed or complemented conventional predictors. Similar comparative logic was also present in Vidiri et al. [26], Mossinelli et al. [29], and Tagliabue et al. [27]. However, this domain was interpreted cautiously because the clinical comparators were heterogeneous and did not consistently represent a fully optimized contemporary clinicopathological framework. Some studies incorporated clinically relevant variables such as depth of invasion, nodal status, margin status, or extranodal/extracapsular extension-related information, whereas others compared imaging-derived models against more limited clinical frameworks, such as clinical T stage or selected clinical variables. Therefore, improved AUC, C-index, or classification performance in combined models was interpreted as evidence of reported complementary performance rather than definitive proof of clinically meaningful incremental value over an AJCC 8th edition–aligned clinical model. Overall, the functional synthesis suggests that MRI-based AI and radiomics may contribute to risk refinement, but their added value requires confirmation against well-specified contemporary clinical and radiological comparators.

The fourth domain, translational maturity and technical implementation, addressed how models were operationalized and how close they came to potential clinical applicability. Most studies relied on manual tumour delineation [26,27,28,29,30,31], indicating continued dependence on expert-driven segmentation workflows. Ren et al. [25] was the only study to implement a fully automated pipeline, combining tumour detection and deep-learning prediction, and therefore represented the highest degree of workflow automation. Validation maturity remained limited overall. Most studies used internal or temporal validation, whereas explicit external validation was clearly represented only by Tagliabue et al. [27] and Ren et al. [25]. Sequence heterogeneity also emerged as a defining feature of this domain. Multiparametric MRI was common, but individual studies differed in the relative value assigned to CE-T1, T2-weighted imaging, DWI, and ADC-derived information. Taken together, this domain shows that although the evidence base is conceptually promising, it remains methodologically heterogeneous and only partially mature for clinical translation.

Overall, the functional meta-synthesis indicates that MRI-based radiomics and artificial intelligence in OTSCC can be organized into four complementary functions: identifying recurrence-prone tumours, expanding prognostic stratification beyond recurrence alone, improving risk estimation when combined with clinical variables, and progressively advancing toward more automated and externally validated workflows. At the same time, the literature remains constrained by retrospective designs, heterogeneous endpoint definitions, and limited external validation, all of which temper immediate generalizability.

### 3.5. Risk of Bias

The risk of bias assessment using PROBAST [18] showed that the overall methodological quality of the included prediction model studies was limited, with most studies judged at high risk of bias overall. The main source of concern was the analysis domain, followed by the participants domain. By contrast, the predictor and outcome domains were generally more robust, because most studies used preoperative MRI-derived variables and objective follow-up-based oncologic endpoints, often with blinded image assessment or standardized outcome definitions. Risk of bias judgments are summarized in Table 3.

Concerns in the participants domain arose mainly from retrospective single-center recruitment, restrictive eligibility criteria, exclusion of patients with incomplete follow-up or incomplete imaging, and selection of surgically treated subgroups only. These features were evident in Cai et al. [30], Yao et al. [28], Vidiri et al. [26], and Wang et al. [31], and may have increased the risk of selection bias and limited representativeness. Ren et al. [25] also used a highly selected cT1-2N0M0 cohort, and disease-free survival was available only for a subset of the full sample, which further constrained applicability for broader prognostic inference. Tagliabue et al. [27] was comparatively stronger in this domain because it validated previous models in an independent multicentric cohort of consecutive patients, although retrospective selection and imaging heterogeneity remained relevant considerations.

The predictor domain was generally judged as low risk, because the MRI-derived predictors were usually obtained from preoperative imaging and, in several studies, segmentation or radiologic assessment was performed in a blinded fashion. This was explicit in Ren et al. [25], Wang et al. [31], and Yao et al. [28]. However, some studies also reported hybrid clinical–radiomic models incorporating post-treatment or pathological variables, particularly Vidiri et al. [26], Mossinelli et al. [29], and Tagliabue et al. [27], which introduced applicability concerns for a review focused on preoperative prognostic modeling, even if this did not necessarily imply high risk of bias in predictor measurement itself.

The outcome domain was mostly low risk, because recurrence, disease-free survival, overall survival, cause-specific mortality, and related events were generally derived from clinical follow-up records and analyzed with standard oncologic definitions. Nevertheless, some studies used broader or less conventional prognostic constructs, such as composite endpoints including recurrence, metastasis, and death, or models primarily designed for nodal prediction with secondary prognostic interpretation. This was particularly relevant for Yao et al. [28], Wang et al. [31], and Ren et al. [25], and produced some applicability concerns when interpreting these models strictly within a recurrence-centered prognostic framework.

The analysis domain was the most problematic across the evidence base. Common limitations included small sample size relative to model complexity, extensive feature extraction with multistep feature selection, reliance on split-sample validation, internal resampling without external confirmation, and a high risk of overfitting. These problems were especially evident in Vidiri et al. [26], Mossinelli et al. [29], Yao et al. [28], Cai et al. [30], and Wang et al. [31]. Even when cross-validation, bootstrap resampling, calibration, or internal testing were reported, these procedures did not fully compensate for the lack of robust external validation in most studies. Ren et al. [25] and Tagliabue et al. [27] were methodologically stronger because they included external validation components, but neither study was clearly low risk overall, since prognostic analyses remained embedded within broader modeling frameworks and retrospective data structures.

Overall, the PROBAST [18] assessment indicates that the current literature is promising but still methodologically fragile. The dominant limitations are consistent with an early translational field: retrospective designs, selective recruitment, high-dimensional modeling with limited event support, heterogeneous validation practices, and only sparse external validation. These factors should be taken into account when interpreting the functional meta-synthesis and the narrative interpretation of secondary prognostic associations.

### 3.6. Certainty of Evidence

The overall certainty of the evidence was judged using a GRADE-informed narrative approach adapted for prognostic model evidence [21,22]. Because the included studies were retrospective prediction model investigations with heterogeneous outcomes, effect measures, MRI sequences, segmentation workflows, and validation strategies, certainty judgments were made at the level of outcome domains rather than for a single pooled effect. The certainty of evidence summary is presented in Table 4.

For the direct recurrence-related outcomes—including loco-regional recurrence, locoregional recurrence-free survival, disease-free survival, and recurrence risk—the certainty of the evidence was judged as low. Although several studies directly addressed recurrence-oriented endpoints [25,26,27,29,30,31], confidence in the evidence was reduced by the predominance of retrospective single-center designs, high or unclear risk of bias in PROBAST, substantial heterogeneity in endpoint definition, and limited external validation. Nonetheless, this domain was supported by relatively coherent clinical direction, because most studies suggested that MRI-derived signatures were associated with poorer recurrence-related outcomes.

For the broader survival-related prognostic outcomes, including overall survival and cause-specific mortality, the certainty was also judged as low. This domain included studies such as Tagliabue et al. [27], Mossinelli et al. [29], Yao et al. [28], and Wang et al. [31], but the evidence was downgraded because of methodological heterogeneity, limited reproducibility across centers, and inconsistent validation depth. Although some studies showed promising discrimination and favorable hazard associations, the number of externally validated models remained very small.

The certainty was judged as very low for composite adverse-event outcomes because this domain was represented by a single study, Yao et al. [28], and because the endpoint structure was not standardized across the evidence base. Although clinically related to prognosis, this outcome was not fully equivalent to recurrence-centered endpoints defined elsewhere in the review.

With respect to the incremental value of MRI-derived models over clinical or clinicopathological frameworks, the certainty was judged as low. Multiple studies reported improved or complementary prognostic performance for combined models [25,26,27,28,29,31], which supports the direction of effect. However, this evidence remained constrained by high-dimensional model construction, limited event counts in several cohorts, heterogeneous comparator structures, and scarce independent external validation.

Secondary DFS or OS associations reported in LNM-focused studies were considered indirect prognostic evidence and were incorporated narratively within the survival-related outcome domain. Because these studies were not primarily designed as recurrence- or survival-prediction models, they did not support a separate quantitative certainty judgment.

Overall, the certainty assessment suggests that the field is promising but still immature. The current literature consistently indicates that MRI-based radiomics and AI may improve preoperative prognostic stratification in OTSCC, yet the strength of inference remains limited by retrospective design, risk of bias, heterogeneity in model construction and endpoints, and the still sparse use of rigorous external validation.

## 4. Discussion

This systematic review synthesized a question not addressed in prior reviews: how preoperative MRI-based radiomics, AI, deep learning, or quantitative MRI-derived models have been evaluated for recurrence and prognostic outcomes in OTSCC. Only seven eligible studies were identified, confirming that this is still an emerging field [25,26,27,28,29,30,31].

Across a summed descriptive sample of 1287 participants, the evidence was uniformly retrospective and heterogeneous in endpoints, MRI sequences, segmentation strategies, model families, and validation design. MRI-derived computational signatures showed plausible prognostic relevance, especially when integrated with clinical variables, but clinical translation remains limited by methodological fragility and sparse external validation.

The review also fills a genuine gap. Earlier reviews focused on diagnostic AI in tongue cancer [12], preoperative prediction of occult lymph node metastasis and DOI [13], broader oral-cancer AI applications for nodal staging or grading [14], recurrence risk by age [15], prognostic biomarkers [16], or preoperative DOI measurement [11]. MRI- and ultrasound-focused syntheses similarly concentrated on imaging-based DOI assessment rather than computational prognostic modelling [32,33,34]. Thus, the novelty of the present review lies in its specific focus on recurrence and survival-oriented MRI-based models in OTSCC.

With respect to outcomes, the present synthesis showed that the included studies collectively covered the main protocol-defined domains, but they did so unevenly. The most direct recurrence-oriented evidence came from Vidiri et al. [26], Mossinelli et al. [29], Tagliabue et al. [27], and Cai et al. [30], which focused on loco-regional recurrence, locoregional recurrence-free survival, recurrence risk, or DFS. These studies are particularly important because they most closely align with the central clinical problem in OTSCC: identifying, before surgery, which tumours are biologically predisposed to relapse despite apparently adequate treatment. Their convergence suggests that preoperative MRI-derived heterogeneity signatures may capture clinically meaningful aspects of tumour aggressiveness that are not fully reflected by conventional staging alone.

At the same time, the evidence was not restricted to recurrence as an isolated endpoint. Yao et al. [28], Mossinelli et al. [29], and Tagliabue et al. [27] contributed to broader prognostic frameworks that incorporated overall survival, cause-specific mortality, or composite adverse events. Ren et al. [25] and Wang et al. [31] were clinically relevant to prognosis, but their primary model target was lymph node metastasis prediction, with DFS or OS associations reported as secondary analyses. Therefore, these two studies were interpreted as indirect prognostic evidence rather than as direct recurrence- or survival-prediction studies. This distinction is important because recurrence was the dominant endpoint in some studies, whereas in others it was embedded within broader constructs or appeared secondarily from models designed for nodal prediction. This heterogeneity complicates direct comparison and explains why quantitative pooling was not methodologically justified.

The breadth of model types included in this review should also be interpreted in this context. Radiomics, conventional machine learning, deep learning, clinicoradiomic nomograms, and quantitative MRI-derived models differ technically and conceptually, and they were not treated as interchangeable methods. They were synthesized together because all attempted to derive prognostic information from preoperative MRI, but their differences in model architecture, feature representation, automation, and validation strategy were preserved within the functional meta-synthesis. Similarly, studies focused primarily on LNM prediction were not used as direct evidence of recurrence or survival prediction; their DFS or OS associations were interpreted only as indirect prognostic signals.

The mixed structure of the reported outcomes also helps clarify how this review differs from previous reviews. Jeong et al. [12] synthesized AI for the diagnosis of tongue cancer, with emphasis on detection, classification, and staging-oriented imaging performance rather than recurrence or long-term prognosis. Ho et al. [13] focused specifically on preoperative prediction of occult lymph node metastasis (OLNM) and DOI in OTSCC, which is clinically adjacent to our question but still conceptually distinct because the primary target is nodal risk or anatomical tumour extent rather than post-treatment recurrence or survival. Mohideen et al. [14] broadened the frame even further to oral and oropharyngeal cancer, including CT, PET/CT, MRI, ultrasound, and histopathology for lymph node metastasis and tumour grading, not recurrence-oriented prognosis. Voizard et al. [11] addressed the reliability of preoperative DOI assessment in OTSCC, and the MRI-focused review by Li et al. [32] reached a similar conclusion regarding imaging-based DOI estimation. These reviews are all relevant comparators, but none specifically synthesized MRI-based radiomics and AI models developed for recurrence and prognostic outcomes in OTSCC.

The functional meta-synthesis was, in methodological terms, the most informative part of the present review because it allowed the evidence to be organized according to what the models were functionally trying to accomplish, rather than forcing heterogeneous studies into an artificial common metric. Four interrelated domains emerged. The first, direct recurrence-oriented modelling, captured the subgroup of studies most explicitly aligned with postoperative failure risk [26,27,29,30]. The second, broader prognostic stratification, included studies in which recurrence was embedded within a wider framework of OS, cause-specific mortality, adverse-event risk, or composite prognosis [27,28,29], while Ren et al. [25] and Wang et al. [31] contributed indirect prognostic information through secondary survival associations from LNM-focused models. The third, incremental value over clinical frameworks, reflected the recurring attempt to show that MRI-derived signatures improve risk estimation beyond conventional clinicopathological variables. The fourth, translational maturity and technical implementation, made visible the distinction between conceptual promise and actual readiness for clinical use.

This domain-based synthesis highlights a major strength of the current evidence base: MRI-based computational biomarkers in OTSCC are not merely imitating conventional staging, but are increasingly being used to refine risk allocation. Several included studies reported that combined clinical–radiomic models outperformed or complemented models based on either imaging or clinical data alone [25,28,31]. This pattern is consistent with broader oncologic imaging literature, where hybrid models integrating radiomics with clinical or pathological variables often perform better than isolated radiomic signatures [35,36,37,38,39]. It also aligns with prior radiomics work in oral cavity and head and neck cancer suggesting that imaging biomarkers are most useful when interpreted within a multivariable prognostic framework rather than as stand-alone predictors [40,41,42].

The apparent incremental value of MRI-derived signatures should also be interpreted with caution. Across the included studies, the clinical-only comparators were heterogeneous and did not consistently represent a fully optimized contemporary clinicopathological framework. DOI and ENE/ECE are central to modern oral-cancer risk stratification and AJCC 8th edition staging, but they were not consistently incorporated into all clinical comparators. In some studies, combined models were compared against limited clinical frameworks, such as clinical T stage or selected clinical variables, whereas other studies included more clinically informative factors such as DOI, nodal status, margin status, or ENE/ECE-related variables. Consequently, a higher AUC, C-index, or accuracy for a combined radiomic–clinical model should not be interpreted automatically as proof of clinically meaningful incremental value. Rather, the current evidence suggests possible complementary prognostic information that requires confirmation against well-specified, AJCC 8th edition-aligned clinical models and expert radiological assessment.

At the same time, the translational domain exposed the immaturity of the field. Most OTSCC studies still depended on manual tumour delineation, centre-specific MRI acquisition, and retrospective single-centre datasets [25,26,27,28,29,30,31]. Only Ren et al. [25] implemented a fully automated workflow, and only Ren et al. [25] and Tagliabue et al. [27] provided clear external validation components. This observation is highly relevant because reproducibility, external validation, and workflow portability are repeatedly identified as the main barriers to clinical adoption in head and neck radiomics [9,10,37,39,41]. Berger et al. [38], for example, showed that radiomics features previously reported as predictive in head and neck cancer did not necessarily generalize well across cohorts, reinforcing the idea that apparent signal strength in one dataset does not guarantee transportability to another clinical setting. Likewise, the broader MRI radiomics review by Corti et al. [40] emphasized that reproducibility problems, heterogeneous image acquisition, and variable preprocessing remain persistent obstacles to meaningful translation.

The decision not to pool the DFS estimates from Ren et al. [25] and Wang et al. [31] was methodologically important. Although both studies reported survival-related associations, their primary purpose was LNM prediction, and their imaging-derived constructs differed substantially. Wang et al. [31] evaluated a peritumoural radiomics signature based on T2-weighted imaging, whereas Ren et al. [25] used an automated deep-learning framework based on ceT1WI and T2WI. These differences in model target, feature representation, and clinical purpose meant that their DFS estimates were better interpreted narratively as secondary prognostic signals, rather than statistically pooled as a common effect.

The PROBAST [18] assessment showed that most included studies were at high overall risk of bias, mainly because of recurring concerns in the Analysis domain. These concerns were particularly relevant to MRI-based radiomics and AI prediction models, where relatively small retrospective cohorts were often combined with high-dimensional feature extraction, multistep feature selection, and complex model-building procedures. In several studies, the number of outcome events appeared limited in relation to the number of candidate predictors or model parameters, raising concern about unstable predictor selection and overfitting; however, formal events-per-variable information was not consistently reported. Most studies relied on split-sample validation, bootstrap procedures, or internal testing, which are useful exploratory methods but do not establish model transportability across institutions, scanners, acquisition protocols, or patient populations. In addition, reporting was often insufficient to determine whether preprocessing, feature selection, hyperparameter tuning, and validation were fully separated, so the possibility of data leakage could not always be excluded. Multicenter image harmonization and standardization were also inconsistently addressed, despite their importance for MRI radiomics reproducibility. These analysis-domain limitations explain why apparently promising discrimination metrics should be interpreted cautiously and why external validation, calibration, transparent model specification, and standardized imaging workflows are essential before clinical implementation can be considered.

The certainty of evidence ranged from low to very low. This was expected because the literature was retrospective, heterogeneous, and only minimally externally validated. Recurrence-related and survival-related domains were judged as low certainty, whereas the composite adverse-event domain was judged as very low because it was represented by single-study evidence and a non-standardized endpoint structure. Secondary DFS or OS associations from LNM-focused studies were incorporated narratively within the survival-related domain and were not treated as a separate quantitative body of evidence. Accordingly, the evidence is promising but not yet strong enough to support routine implementation [42,43,44].

The present review has several limitations that should be acknowledged. First, the number of included studies was small, and the evidence was restricted to retrospective cohorts from only two countries. Second, the primary studies were highly heterogeneous, which limited formal quantitative synthesis and required an interpretive rather than metric-driven analytical strategy. Third, because some studies combined recurrence with broader prognostic constructs or reported recurrence only secondarily, the conceptual boundaries of the included outcome domains were necessarily broader than they would be in an idealized, purely recurrence-specific evidence base. Fourth, secondary DFS or OS associations from LNM-focused models were available in only a small number of studies and were interpreted narratively because they were not clinically or methodologically comparable enough for pooling. Fifth, although the revised eligibility criteria focused on squamous cell carcinoma of the oral/mobile tongue and excluded studies with reported base-of-tongue or other oropharyngeal involvement, anatomical site definitions were not completely explicit in all primary studies. In particular, some articles used broader TSCC or tongue-cancer terminology, or did not report base-of-tongue involvement rather than actively excluding it, which means that residual anatomical-site uncertainty cannot be fully ruled out. Finally, although the review deliberately focused on MRI-based models to preserve conceptual coherence and fit the oncologic imaging question, this also means that evidence from CT-, PET-, or histopathology-based oral oncology AI studies was considered only for contextual comparison rather than direct synthesis.

These limitations, however, should be weighed against the strengths of the review. To our knowledge, this is the first systematic review specifically dedicated to MRI-based radiomics and AI models for recurrence and prognostic outcomes in OTSCC. It therefore addresses a genuine gap left by existing systematic reviews, which have focused on diagnosis [12], OLNM/DOI prediction [13], oral-cancer nodal metastasis and grading across broader populations [14], or preoperative DOI measurement [11,32]. The review did not force heterogeneous studies into an artificial pooled summary, but instead used a functional meta-synthesis to preserve clinical interpretability while making methodological uncertainty explicit. The explicit use of PROBAST [18] and a GRADE [21,22]-informed approach further strengthens the credibility of the conclusions by making uncertainty visible rather than obscuring it. In addition, the review remains tightly anchored in MRI-based oncologic imaging, which gives it a clearer translational identity within precision head and neck oncology.

From a clinical perspective, the available evidence should be interpreted as preliminary and hypothesis-generating. The current findings do not demonstrate that MRI-based radiomics or AI can reliably predict recurrence or survival in routine OTSCC care. Rather, early retrospective studies suggest that MRI-derived computational signatures may be associated with recurrence-related or survival-related outcomes and may provide complementary information when combined with clinical or clinicopathological variables [25,26,27,28,29,30,31]. At this stage, these models are best understood as investigational tools for risk exploration rather than as validated decision-making systems. Their most plausible future application is in multidisciplinary treatment planning, where externally validated and well-calibrated MRI-derived signatures could eventually complement DOI, nodal status, margin expectations, or expert radiological interpretation when planning surgery, neck management, postoperative surveillance, and adjuvant treatment discussions. This is particularly relevant in OTSCC, where local control, regional failure, and disease-specific death may diverge despite apparently similar clinicopathological profiles [2,3,4].

However, the review does not support immediate routine adoption. Before these models can be integrated into clinical pathways, they need to demonstrate reproducibility across scanners and institutions, robust calibration, clinically meaningful net benefit, and incremental value over expert radiological assessment. The problem is therefore not simply to improve discrimination, but to establish trustworthiness and transportability. The literature reviewed by Corti et al. [40] and Valizadeh et al. [38] suggests that this transition from proof-of-concept to deployment remains the principal challenge in head and neck radiomics more broadly. Our review indicates that OTSCC is no exception.

Future studies should prioritize prospective multicentre validation, standardized recurrence and survival endpoints, transparent calibration reporting, and direct comparison against expert radiological assessment. Greater methodological consistency would also determine whether future quantitative synthesis is clinically and statistically appropriate. In parallel, automated or semi-automated workflows should be tested across heterogeneous scanners and institutions to determine whether efficiency can be gained without sacrificing reproducibility. Overall, MRI-based computational biomarkers in OTSCC are promising, but the next phase of research must be translational rather than merely exploratory.

## 5. Conclusions

MRI-based radiomics and artificial intelligence may have preliminary potential to inform preoperative prognostic stratification in oral tongue squamous cell carcinoma, particularly for recurrence-related risk refinement and survival-oriented assessment beyond conventional clinicopathological variables. At the same time, the current evidence base remains methodologically limited by retrospective designs, heterogeneous imaging workflows and outcome definitions, small single-centre cohorts, sparse external validation, and low to very low certainty of evidence. The functional meta-synthesis suggests that these heterogeneous approaches can be organized into complementary roles in direct recurrence-oriented modeling, broader prognostic stratification, integration with clinical frameworks, and translational workflow development. However, secondary DFS or OS associations from LNM-focused models should be interpreted as indirect prognostic signals rather than confirmatory evidence of validated recurrence or survival prediction. Overall, these findings support the preliminary clinical promise of MRI-derived computational biomarkers in oncologic imaging, but this review does not establish clinical utility or readiness for routine implementation. Larger prospective multicenter studies, standardized methodology, robust external validation, transparent reporting, and direct assessment of incremental value over expert radiological and clinicopathological evaluation are required before these models can be considered for clinical decision-making.

## Figures and Tables

**Table 1:** Descriptive characteristics of included studies.

Study	Design and Country	Sample	Anatomical Site Definition Reported	MRI-Based Approach	Outcomes and Validation
Ren et al., 2026, China [25]	Retrospective, two-center model development and validation study	348	Explicit OTSCC; early-stage oral tongue squamous cell carcinoma	Fully automated MRI deep learning with YOLOv8 tumor detection and encoder-based prediction from ceT1WI and T2WI	Primary focus on LNM prediction with recurrence-risk relevance; DFS association; validation, internal testing, and external testing cohorts
Vidiri et al., 2025, Italy [26]	Retrospective single-center study	92	Explicit OTSCC; oral tongue squamous cell carcinoma	MRI radiomics from preoperative post-contrast high-resolution MRI difference volumes	Loco-regional recurrence prediction; stratified training/validation split
Tagliabue et al., 2024, Italy [27]	Retrospective multicentric external validation study	112	Explicit OTSCC; oral tongue squamous cell carcinoma	External validation of previously published MRI-radiomic models based on CE-T1 and ADC	OS, LRRFS, CSM; external validation across institutions
Yao et al., 2024, China [28]	Retrospective single-center study	232	No base-of-tongue involvement reported	MRI radiomics from T2WI, CE-T1WI, DWI, and ADC integrated with clinical factors	OS and adverse-event risk; temporal training/test split
Mossinelli et al., 2023, Italy [29]	Retrospective single-center study	79	Explicit OTSCC/mobile tongue cancer	MRI radiomics from T1, T2, DWI, and ADC with clinical–radiomic modeling	LRR, CSM, OS; internal validation and bootstrap
Cai et al., 2022, China [30]	Retrospective cohort study	188	Explicit OTSCC; oral tongue squamous cell carcinoma	Quantitative MRI-derived ADC ratio prognostic model and nomogram	Recurrence and DFS; internal validation
Wang et al., 2022, China [31]	Retrospective study with training/testing cohorts	236	No base-of-tongue involvement reported	MRI radiomics with peritumoral extensions and clinicopathological integration on T2WI	LNM prediction with prognostic association for DFS and OS; independent testing cohort

MRI, magnetic resonance imaging; YOLOv8, You Only Look Once version 8; ceT1WI, contrast-enhanced T1-weighted imaging; T2WI, T2-weighted imaging; LNM, lymph node metastasis; DFS, disease-free survival; CE-T1, contrast-enhanced T1-weighted imaging; ADC, apparent diffusion coefficient; OS, overall survival; LRRFS, locoregional recurrence-free survival; CSM, cause-specific mortality; DWI, diffusion-weighted imaging; T1, T1-weighted imaging; T2, T2-weighted imaging; LRR, loco-regional recurrence.

**Table 2:** Contribution of the included studies to the main and additional outcomes.

Study	Main Outcomes	Additional Outcomes	Key Reported Metrics
Ren et al., [25]	Primary LNM prediction; secondary DFS-linked prognostic association	Validation across multiple cohorts; automated workflow; incremental value over clinical T stage	AUC 0.871/0.776/0.909 for automated DL nomogram; HR for DFS 1.82
Vidiri et al., [26]	Loco-regional recurrence prediction	Comparison of clinical, radiomic, and combined models	Accuracy 0.79 in training and 0.74 in validation for the radiomic-only model
Tagliabue et al., [27]	LRRFS	OS, CSM, external validation, sequence-specific comparison	CE-T1 validation C-indexes 0.61/0.59/0.64 for pretreatment OS/LRRFS/CSM and 0.65/0.69/0.70 for posttreatment models
Yao et al., [28]	Composite recurrence-related adverse events	OS; nomogram validation; sequence-specific feature reduction	C-index 0.86/0.81; AUC 0.898/0.875
Mossinelli et al., [29]	LRR	CSM, OS, sequence comparison, incremental value over clinical models	ADC C-index 0.98 for LRR, 0.86 for CSM, 0.84 for OS
Cai et al., [30]	Recurrence, DFS	Calibration and internal validation of quantitative MRI-derived nomogram	Sensitivity 71.1%; specificity 81.0%; C-index > 0.70
Wang et al., [31]	Primary LNM prediction; secondary DFS/OS prognostic association	OS; peritumoral modeling; comparison against MRI-reported LN status and clinicopathological models	Testing AUC 0.872 for CRprim + 10; HR 5.250 for DFS and 17.464 for OS

DFS, disease-free survival; DL, deep learning; LRR, loco-regional recurrence; LRRFS, locoregional recurrence-free survival; OS, overall survival; CSM, cause-specific mortality; AUC, area under the receiver operating characteristic curve; HR, hazard ratio; ADC, apparent diffusion coefficient; CE-T1, contrast-enhanced T1-weighted imaging; LN, lymph node.

**Table 3:** PROBAST-based risk of bias assessment of included studies.

Study	Participants	Predictors	Outcome	Analysis	Overall Risk of Bias	Main Applicability Concerns
Ren et al., [25]	High	Low	Low	High	High	Restricted to cT1-2N0M0 early-stage patients; prognostic interpretation secondary to an LNM-focused model
Vidiri et al., [26]	High	Low	Low	High	High	Small monocentric cohort; inclusion of post-treatment hybrid models alongside pre-treatment models
Tagliabue et al., [27]	Low	Unclear	Low	Unclear	Unclear	External validation study, but with heterogeneous multicenter imaging sources and some limited external imaging detail
Yao et al., [28]	High	Low	Unclear	High	High	Composite adverse-event/OS framework rather than pure recurrence-oriented prediction
Mossinelli et al., [29]	High	Unclear	Low	High	High	Small single-center cohort; inclusion of post-treatment clinical–radiomic models
Cai et al., [30]	High	Low	Low	High	High	Surgically treated retrospective cohort only; internal validation only
Wang et al., [31]	High	Low	Unclear	High	High	Primary model focused on LNM, with prognostic interpretation for DFS/OS added secondarily

PROBAST, Prediction model Risk Of Bias ASsessment Tool; LNM, lymph node metastasis; DFS, disease-free survival; OS, overall survival; cT, clinical tumor stage; N0, no regional lymph node metastasis; M0, no distant metastasis. High risk of bias in the PROBAST analysis domain was mainly driven by recurring limitations typical of radiomics and AI prediction model studies: small retrospective cohorts relative to model complexity; limited event support for high-dimensional feature extraction; multistep feature selection before or during model development; reliance on split-sample, bootstrap, or internal validation without robust external confirmation; incomplete reporting of calibration, model updating, and handling of missing data; and insufficient information to fully exclude overfitting or data leakage during feature selection, hyperparameter tuning, and validation. These concerns were interpreted according to the information reported in each primary study and were not treated as evidence of definite methodological misconduct.

**Table 4:** GRADE-informed certainty of evidence summary for the main prognostic domains.

Outcome Domain	Contributing Studies	Main Concerns	Certainty of Evidence
Recurrence-related outcomes, including loco-regional recurrence, LRRFS, DFS, and recurrence risk	[25,26,27,29,30,31]	High/unclear risk of bias in most studies; retrospective designs; heterogeneous recurrence definitions; limited external validation; imprecision in several cohorts	Low
Survival-related outcomes, including OS, CSM, and DFS when survival-oriented	[25,27,28,29,30,31]	Methodological heterogeneity; variable model types and sequences; mostly internal validation; limited reproducibility across centers	Low
Composite adverse-event outcomes	[28]	Single-study evidence; indirectness due to non-standardized adverse-event outcome structure; limited external validation	Very low
Incremental value of MRI-derived models over clinical or clinicopathological models	[25,26,27,28,29,31]	Heterogeneous comparators; risk of overfitting; limited external validation; high-dimensional modeling with variable event support	Low

GRADE [21,22] was applied in a narrative, outcome-domain-oriented manner, adapted to the prognostic model evidence base and the marked methodological heterogeneity of the included studies. LRRFS, locoregional recurrence-free survival; DFS, disease-free survival; OS, overall survival; CSM, cause-specific mortality; MRI, magnetic resonance imaging.

## Data Availability

The original contributions presented in this study are included in the article/Supplementary Material. Further inquiries can be directed to the corresponding author.

## Supplementary Materials

The following supporting information can be downloaded at https://www.mdpi.com/article/10.3390/medsci14020332/s1. PRISMA 2020 Checklist. Table S1. Full search strategies used in PubMed, Scopus, and Embase. Table S2. Full-text articles excluded, with reasons.
