# Supplementary material for: MRI-Based Radiomics and Artificial Intelligence for Prediction of Recurrence and Prognostic Outcomes in Oral Tongue Squamous Cell Carcinoma: A Systematic Review with Functional Meta-Synthesis
Source: Med Sci (Basel). 2026 Jun 19;14(2):332. doi: 10.3390/medsci14020332 (PMC13302954; doi:10.3390/medsci14020332)
Supplement: Supplementary file 1 [file medsci-14-00332-s001.zip › Supplementary Table S1 Search Strategies .pdf]

Supplementary Table S1. Full search strategies used in PubMed, Scopus, and Embase

Database-specific syntax was adapted during protocol development and piloting. PubMed and Scopus strategies reproduce the finalized pilot searches. The Embase strategy was simplified and adapted to the syntax and retrieval behavior of the platform used, prioritizing stable and focused retrieval over a more complex syntax that yielded null or uninformative results.

Database	Search string
PubMed	(<br>(<br>"Tongue Neoplasms"[Mesh]<br>OR "tongue squamous cell carcinoma"[Title/Abstract]<br>OR "oral tongue squamous cell carcinoma"[Title/Abstract]<br>OR OTSCC[Title/Abstract]<br>OR TSCC[Title/Abstract]<br>OR "tongue cancer"[Title/Abstract]<br>OR "oral tongue cancer"[Title/Abstract]<br>)<br>AND<br>(<br>"Magnetic Resonance Imaging"[Mesh]<br>OR MRI[Title/Abstract]<br>OR "magnetic resonance imaging"[Title/Abstract]<br>OR "MR imaging"[Title/Abstract]<br>OR "contrast-enhanced MRI"[Title/Abstract]<br>OR "multiparametric MRI"[Title/Abstract]<br>)<br>AND<br>(<br>radiomic*[Title/Abstract]<br>OR "texture analysis"[Title/Abstract]<br>OR "machine learning"[Title/Abstract]<br>OR "deep learning"[Title/Abstract]<br>OR "artificial intelligence"[Title/Abstract]<br>OR "deep neural network"[Title/Abstract]<br>OR "neural network*"[Title/Abstract]<br>OR classifier[Title/Abstract]<br>OR nomogram[Title/Abstract]<br>OR "prediction model"[Title/Abstract]<br>OR "predictive model"[Title/Abstract]<br>)<br>AND<br>(<br>recurren*[Title/Abstract]<br>OR "loco-regional recurrence"[Title/Abstract]<br>OR "locoregional recurrence"[Title/Abstract]<br>OR relapse[Title/Abstract]<br>OR "recurrence risk"[Title/Abstract]<br>OR prognosis[Title/Abstract]<br>OR prognostic[Title/Abstract]<br>OR survival[Title/Abstract]<br>OR outcome[Title/Abstract]<br>OR risk[Title/Abstract]

Database	Search string
	)<br>)
Scopus	TITLE-ABS-KEY(<br>(<br>"oral tongue squamous cell carcinoma"<br>OR OTSCC<br>OR "tongue squamous cell carcinoma"<br>OR TSCC<br>OR "oral tongue cancer"<br>OR "tongue cancer"<br>)<br>AND<br>(<br>MRI<br>OR "magnetic resonance imaging"<br>OR "MR imaging"<br>OR "diffusion-weighted imaging"<br>OR DWI<br>OR ADC<br>OR "contrast-enhanced MRI"<br>)<br>AND<br>(<br>radiomic*<br>OR "texture analysis"<br>OR "machine learning"<br>OR "deep learning"<br>OR "artificial intelligence"<br>OR "prediction model"<br>OR "predictive model"<br>OR nomogram<br>OR "imaging biomarker*"<br>OR classifier<br>)<br>AND<br>(<br>recurren*<br>OR relapse<br>OR "loco-regional recurrence"<br>OR locoregional<br>OR "locoregional recurrence-free survival"<br>OR LRRFS<br>OR prognosis<br>OR prognostic<br>OR "disease-free survival"<br>OR DFS<br>OR "overall survival"<br>OR OS<br>OR "cause-specific mortality"<br>OR CSM

Database	Search string
	OR outcome*<br>OR survival<br>)<br>)
Embase (Ovid)	(tongue NEAR/3 cancer):ti,ab<br>AND<br>(MRI OR ADC OR DWI):ti,ab<br>AND<br>(radiomics OR nomogram OR prognosis OR recurrence):ti,ab
