# Supplementary material for: MRI-Based Radiomics and Artificial Intelligence for Prediction of Recurrence and Prognostic Outcomes in Oral Tongue Squamous Cell Carcinoma: A Systematic Review with Functional Meta-Synthesis
Source: Med Sci (Basel). 2026 Jun 19;14(2):332. doi: 10.3390/medsci14020332 (PMC13302954; doi:10.3390/medsci14020332)
Supplement: Supplementary file 1 [file medsci-14-00332-s001.zip › Supplementary Table S2 Full-text articles excluded, with reasons.pdf]

Supplementary Table S2. Full-text articles excluded, with reasons

Study	Main reason for exclusion
Wang X, Fu S, Ren J, Tan P, Wei T, Liu Y, et al. 2026. *Deep learning radiomics based on multimodal MRI for preoperative prediction of N stage in tongue squamous cell carcinoma: a multicenter study.*	Excluded because the study focused on preoperative nodal staging (N stage) rather than recurrence- or survival-related prognostic outcomes.
Gang Q, Feng J, Chen B, Zhang N, Zhang K. 2026. *TensorFlow-based MobileNetV2 U-Net tumor segmentation and multiparametric MRI radiomics for predicting cervical lymph node metastasis in oral tongue squamous cell carcinoma.*	Excluded because the model was developed for cervical lymph node metastasis prediction, without reporting recurrence- or survival-related prognostic endpoints.
Jeong S, Choi HI, Yang KI, Kim JS, Ryu JW, Park HJ. 2025. *Artificial Intelligence in the Diagnosis of Tongue Cancer: A Systematic Review with Meta-Analysis.*	Excluded because it was a systematic review with meta-analysis, whereas the present review included only original primary studies.
Li Y, Huang N, Wang L, Xiao H, Chen W, Xing Y, et al. 2025. *An interpretable machine learning model using SHapley Additive exPlanations for preoperative cervical lymph node metastasis risk stratification in tongue squamous cell carcinoma: a multicenter study.*	Excluded because the study addressed preoperative cervical lymph node metastasis risk stratification, not recurrence or survival-related prognostic modeling.
Hui Leonard TT, Yang MC, See A, Huang Y, Tsai TY, Lee CY, et al. 2025. *Tumor-width-to-tongue-width ratio: A novel predictor of cervical lymph node metastasis and prognosis in squamous cell carcinoma of the tongue.*	Excluded because it was based on a conventional MRI-derived morphologic ratio and clinicopathological nomogram, rather than an MRI-based radiomics, AI, deep learning, or quantitative MRI-derived prognostic model aligned with the review focus.
Li W, Li Y, Wang L, Yang M, Iikubo M, Huang N, et al. 2025. *Evaluating fusion models for predicting occult lymph node metastasis in tongue squamous cell carcinoma.*	Excluded because the primary outcome was occult lymph node metastasis, with no direct modeling of recurrence- or survival-related prognostic outcomes.
Oki A, Nakamura S, Sakamoto J, Watanabe H, Miura M. 2025. *Can MRI radiomics predict neck metastasis at initial diagnosis in patients with squamous cell carcinoma of the tongue?*	Excluded because the study focused on neck metastasis at initial diagnosis, not on recurrence, disease-free survival, or other prognostic outcomes after treatment.
See A, Yang MC, Huang Y, Tsai TY, Lee CY, Huang PH, et al. 2025. *Radiological Sublingual Space Invasion in Tongue Squamous Cell Carcinoma: Clinicopathological Associations and Impact on Survival.*	Excluded because it evaluated a conventional MRI-assessed radiological feature (sublingual space invasion) and associated nomogram, rather than an MRI-based radiomics/AI or quantitative MRI signal-derived prognostic model within the predefined review scope.

Study	Main reason for exclusion
Gang Q, Feng J, Kauczor HU, Zhang K. 2024. *Predicting nodal metastasis progression of oral tongue cancer using a hidden Markov model in MRI.*	Excluded because the study modeled nodal metastasis progression, not recurrence- or survival-related prognostic outcomes.
Liu S, Zhang A, Xiong J, Su X, Zhou Y, Li Y, et al. 2024. *The application of radiomics machine learning models based on multimodal MRI with different sequence combinations in predicting cervical lymph node metastasis in oral tongue squamous cell carcinoma patients.*	Excluded because the study focused on cervical lymph node metastasis prediction, without reporting recurrence or survival-related prognostic endpoints.
Saenthaveesuk P, Yang L, Zeng B, Xu M, Young S, Liao G, et al. 2021. *Development and validation of multiparametric MRI-based nomogram for predicting occult metastasis risk in early tongue squamous cell carcinoma.*	Excluded because the study focused on occult cervical metastasis risk, not on recurrence- or survival-related prognostic outcomes as required for the review.
Ren J, Qi M, Yuan Y, Tao X. 2021. *Radiomics of apparent diffusion coefficient maps to predict histologic grade in squamous cell carcinoma of the oral tongue and floor of mouth: a preliminary study.*	Excluded because the endpoint was histologic grade, not recurrence or prognosis, and because the cohort included oral tongue and floor-of-mouth tumors combined, without a tongue-specific recurrence/prognostic model.
Liu J, Song L, Zhou J, Yu M, Hu Y, Zhang J, et al. 2023. *Prediction of Prognosis of Tongue Squamous Cell Carcinoma Based on Clinical MR Imaging Data Modeling*	Cohort included base-of-tongue tumors and did not provide extractable oral/mobile tongue-specific prognostic model results.
